# New Insights into the Mediation of Biofilm Formation by Three Core Extracellular Polysaccharide Biosynthesis Pathways in *Pseudomonas aeruginosa*

**DOI:** 10.3390/ijms26083780

**Published:** 2025-04-17

**Authors:** Qianhui Liu, Qian Wu, Jiawen Liu, Tianming Xu, Jing Liu, Qin Wu, Pradeep K. Malakar, Yongheng Zhu, Yong Zhao, Zhaohuan Zhang

**Affiliations:** 1 College of Food Science and Technology, Shanghai Ocean University, 999# Hu Cheng Huan Road, Shanghai 201306, China; m220351069@st.shou.edu.cn (Q.L.); d220300083@st.shou.edu.cn (Q.W.); liujw0612@163.com (J.L.); d230300096@st.shou.edu.cn (T.X.); d210300079@st.shou.edu.cn (J.L.); 18256356162@163.com (Q.W.); pkmalakar@shou.edu.cn (P.K.M.); yh-zhu@shou.edu.cn (Y.Z.); 2 International Research Center for Food and Health, Shanghai Ocean University, 999# Hu Cheng Huan Road, Shanghai 201306, China; 3 Laboratory of Quality & Safety Risk Assessment for Aquatic Products on Storage and Preservation (Shanghai), Ministry of Agriculture and Rural Affairs, 999# Hu Cheng Huan Road, Shanghai 201306, China; 4 Shanghai Engineering Research Center of Aquatic-Product Processing & Preservation, 999# Hu Cheng Huan Road, Shanghai 201306, China

**Keywords:** *Pseudomonas aeruginosa*, biofilm, alginate biosynthetic system, Psl biosynthetic system, Pel biosynthetic system

## Abstract

*Pseudomonas aeruginosa* biofilms, driven by extracellular polysaccharides (EPSs), exacerbate pathogenicity and drug resistance, posing critical threats to public health. While EPS biosynthesis pathways are central to biofilm formation, their distinct contributions and regulatory dynamics remain incompletely understood. Here, we systematically dissect the roles of three core EPS pathways—Psl, Pel, and alginate—in biofilm architecture and function using multi-omics approaches. Key findings reveal Psl as the dominant regulator of biofilm elasticity and thickness, with its deletion disrupting chemotaxis, quorum sensing, and 3′,5′-Cyclic GMP (c-di-GMP)/amino acid metabolism. Pel redundantly enhances biofilm biomass, but elevates flagellar synthesis efficiency when Psl is absent. Alginate exhibited negligible transcriptional or metabolic influence on biofilms. These insights clarify hierarchical EPS contributions and highlight Psl as a priority target for therapeutic strategies to dismantle biofilm-mediated resistance.

## 1. Introduction

*Pseudomonas aeruginosa* is a common opportunistic pathogen that not only contributes to significant water pollution, but also causes various chronic or acute diseases in humans [1]. This organism readily forms biofilms and is widely regarded as a model strain for biofilm research [2]. Biofilms are multicellular aggregates composed of bacterial cells and their secretions, adhering to biological or non-biological surfaces [3]. These structures enhance the pathogenicity and antibiotic resistance of bacteria, posing serious threats to environmental safety and public health [4]. Extracellular polysaccharides play an essential role in *Pseudomonas* biofilm, enhancing bacterial invasion of host cells and resistance to antibiotics [4]. Research has identified three core extracellular polysaccharides directly associated with *Pseudomonas* biofilm formation: Alginate, Psl, and Pel. These polysaccharides are synthesized and transported through the alginate biosynthesis pathway, Psl biosynthesis pathway, and Pel biosynthesis pathway, respectively [5]. A more in-depth investigation into the synthesis and transport mechanism of these core extracellular polysaccharides in *P. aeruginosa* biofilms will significantly enhance our understanding of the biofilm formation processes in this pathogen.

Alginate is an anionic polysaccharide composed of β-D-mannuronic acid and its C5 isomer α-L-guluronic acid, frequently observed in the airways of patients with pulmonary cystic fibrosis [1]. Its biosynthesis and transport are mediated by proteins encoded by the *alg* gene cluster, among which Alg8, a glycosyltransferase, plays an important role. Experimental evidence indicates that Alg8 is essential for alginate biosynthesis, with its knockout of *alg8* resulting in defective alginate production in *P. aeruginosa* FRD1, which typically overexpresses alginate [6]. Psl is a pentasaccharide (five monosaccharide sugars) containing D-mannose, D-glucose, and L-rhamnose. The *psl*-dependent polysaccharide consists of repeated units of Psl [7]. It serves as the primary extracellular polysaccharide of *P. aeruginosa* PAO1, a model strain of *P. aeruginosa*, and contributes to biofilm elasticity [8,9,10]. Psl synthesis is directed by the *pslA-O* gene cluster, where PslA is an inner membrane protein with polyisoprene glycosyltransferase activity, and PslB is a bifunctional enzyme involved in the production of GDP-mannose (GDP-Man), a glyconucleotide precursor of Psl polysaccharide. The absence of *pslAB* causes significant disruption to the PAO1 biofilm [11,12,13]. Pel is a cationic extracellular polysaccharide composed of partially acetylated N-acetylglucosamine and N-acetylglucosamine [14], forming a thin film at the air–water interface of *P. aeruginosa*. This film aids in antibiotic resistance and contributes to the structural and protective aspects of the biofilm matrix. The biosynthesis of Pel is governed by the *pelA-G* gene cluster, with PelF functioning as a glycosyltransferase responsible for transferring sugar units from sugar-nucleotides to Pel polysaccharides. The absence of the *pelF* gene results in the inability to synthesize Pel polysaccharides [15]. Despite these insights, the core mechanisms underlying extracellular polysaccharide synthesis and transport in *P. aeruginosa* biofilm remain incompletely understood, with many transport mechanisms still not fully elucidated.

The integration of transcriptomics and metabolomics involves combining data on gene expression (transcriptome) and metabolic profiles (metabolome) to comprehensively elucidate the regulatory mechanisms underlying complex physiological processes in biological systems. In biofilm research, this multi-omics approach reveals the intrinsic connections between gene expression dynamics and metabolic reprogramming during biofilm formation, providing critical insights into the molecular mechanisms of biofilms and supporting the development of targeted control strategies [16,17,18,19]. Despite these advancements, multi-omics studies specifically investigating the synthesis and transport of extracellular polysaccharides in the core of *P. aeruginosa* biofilms remain underexplored. Employing multi-omics techniques could significantly enhance our understanding of the mechanisms governing core extracellular polysaccharide synthesis and transport in *P. aeruginosa* biofilms.

Given the key roles of Alg8, PslAB, and PelF in extracellular polysaccharide biosynthesis, this study compares the wild-type PAO1 strain with three mutant strains of *P. aeruginosa* PAO1. Specifically, the strains PAO1-Δ*pelF*, PAO1-Δ*pslAB*, and PAO1-Δ*alg8* were investigated. Initially, crystal violet staining, quartz crystal microbalance with dissipation, and microscopic imaging techniques were employed for multidimensional characterization of the biofilms formed by these strains. Subsequently, a combined transcriptomics and metabolomics approach was used to analyze the internal regulatory mechanisms governing biofilm formation in *P. aeruginosa*. This method allowed for a comprehensive examination of the roles of three core extracellular polysaccharide biosynthetic systems, addressing phenotypic changes, transcriptional regulation, and metabolic variations. This study provides a thorough and systematic elucidation of the mechanisms underlying biofilm formation mediated by these core biosynthetic systems.

## 2. Results

### 2.1. Phenotypic Changes in P. aeruginosa Biofilm Mediated by Core Extracellular Polysaccharide Biosynthesis Pathways

**Figure 1:**
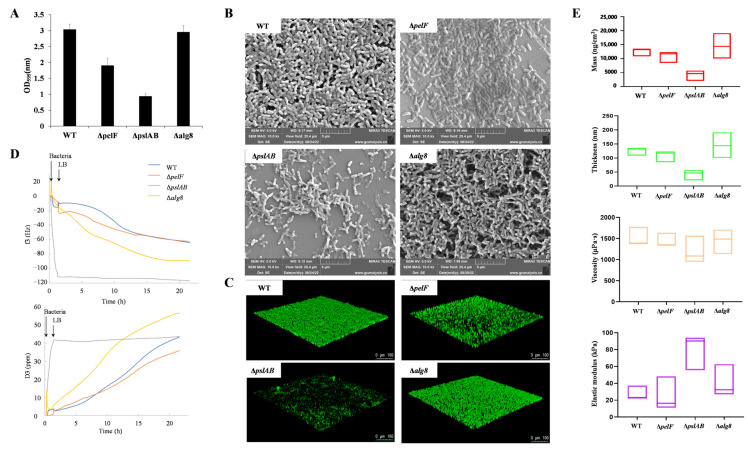
Phenotypic characterization of biofilms. (**A**) Determination of crystal violet staining on *P. aeruginosa* PAO1 biofilm. (**B**) SEM image of *P. aeruginosa* PAO1 biofilm. (**C**) CLSM image of *P. aeruginosa* PAO1 biofilm. (**D**) Representative frequency shifts (f) and dissipation shifts. (**D**) of the third overtone for *P. aeruginosa* PAO1 biofilm determined by QCM-D. (**E**) QCM-D characterization for biofilm parameters of *P. aeruginosa* PAO1 after 20 h. (Note: red represents mass; green represents thickness; yellow represents viscosity; purple represents elastic modulus).

Crystal violet staining was used to evaluate biofilm formation in *P. aeruginosa* strains PAO1-WT, PAO1-Δ*pelF*, PAO1-Δ*pslAB*, and PAO1-Δ*alg8* after 24 h of cultivation, as shown in Figure 1A. The strain PAO1-Δ*pelF* formed approximately one-third of the biofilm formation observed in the strain PAO1-WT. Conversely, the strain PAO1-Δ*pslAB* demonstrated a more substantial reduction, with biofilm levels decreasing to less than one-third of those in PAO1-WT. No significant differences in biofilm formation were observed between the PAO1-Δ*alg8* and PAO1-WT strains.

To visually demonstrate the effects of three extracellular polysaccharide biosynthesis pathways on biofilm morphology and content, SEM was employed to examine the biofilms of strains PAO1-WT, PAO1-Δ*pelF*, PAO1-Δ*pslAB*, and PAO1-Δ*alg8* (Figure 1B). Observations revealed that the biofilm of strain PAO1-Δ*pslAB* differed significantly from that of PAO1-WT, with the latter exhibiting a denser biofilm structure, while PAO1-Δ*pslAB* showed a marked reduction in biofilm formation. The strain PAO1-Δ*pelF* also demonstrated a notably reduced biofilm compared to strain PAO1-WT. Although no significant difference in biofilm content was observed between strains PAO1-Δ*alg8* and PAO1-WT, the strain PAO1-Δ*alg8* biofilm lacked filamentous material external to the bacterial cells, potentially attributable to the absence of alginate extracellular polysaccharides.

Subsequently, CLSM was employed to observe and quantify the biofilms of strains PAO1-WT, PAO1-Δ*pelF*, PAO1-Δ*pslAB*, and PAO1-Δ*alg8* (Figure 1C and Table S1). The strain PAO1-Δ*pelF* demonstrated a reduction in both biofilm amount and thickness, although its roughness was similar to that of PAO1-WT. The strain PAO1-Δ*pslAB* exhibited approximately half the biofilm biomass of PAO1-WT and a significant decrease in thickness, with a more than twofold increase in roughness. In contrast, the biofilm characteristics of strain PAO1-Δ*alg8* closely resembled those of PAO1-WT.

The real-time monitoring of biofilms formed by strains PAO1-WT, PAO1-Δ*pelF*, PAO1-Δ*pslAB*, and PAO1-Δ*alg8* was conducted using QCM-D (Figure 1D). Following a one-hour inoculation with bacterial solutions and subsequent continuous perfusion with LB medium, strain PAO1-WT exhibited a negative trend in the frequency shift parameter (f3), which decreased over time. Concurrently, the dissipation factor (D3) showed a sustained increase, indicating a rise in dissipation. These results suggest that the PAO1-WT biofilm behaves as a flexible membrane. Over the course of the experiment, the biofilm progressively matured, with a continuous increase in mass. In contrast, the (f3) and (D3) curves for strain PAO1-Δ*pslAB* diverged significantly from those of the other strains. In the initial 20 h of biofilm development, changes in (f3) and (D3) for PAO1-Δ*pslAB* were minimal, with (D3) remaining relatively stable post-inoculation, indicative of slow biofilm growth and modest mass increase. 

Using QSense Dind v1.0.0, we analyzed various biofilm indices grown on silica chips for 20 h (Figure 1E). The mass and thickness of the PAO1-Δ*pslAB* biofilm were approximately one-third of those of the PAO1-WT biofilm, while the PAO1-Δ*pelF* biofilm exhibited a slight reduction in mass and thickness. Viscosity measurements revealed no significant differences among the strains compared to PAO1-WT. Notably, the elastic modulus of the PAO1-Δ*pslAB* biofilm was significantly higher than that of the other strains, nearly quadrupling in value. This increased elastic modulus corresponds to greater rigidity, suggesting that the PAO1-Δ*pslAB* biofilm exhibits enhanced rigidity and reduced elasticity. These findings indicate that the Psl biosynthetic system plays a crucial role in modulating biofilm properties, contributing to a higher degree of elasticity in biofilms influenced by this system.

Therefore, the Psl biosynthesis pathway is essential for the formation of *P. aeruginosa* biofilm; it increases both the quantity and thickness of the biofilm while enhancing its elasticity through the production of Psl extracellular polysaccharides. The Pel biosynthesis pathway plays a role in *P. aeruginosa* biofilm formation, providing a supplementary function that reinforces the biofilm structure. In contrast, the alginate biosynthetic system has the least impact on effect on the formation of PAO1 biofilm.

### 2.2. Transcription Regulation of P. aeruginosa Biofilm Mediated by Core Extracellular Polysaccharide Biosynthesis Pathways

**Figure 2:**
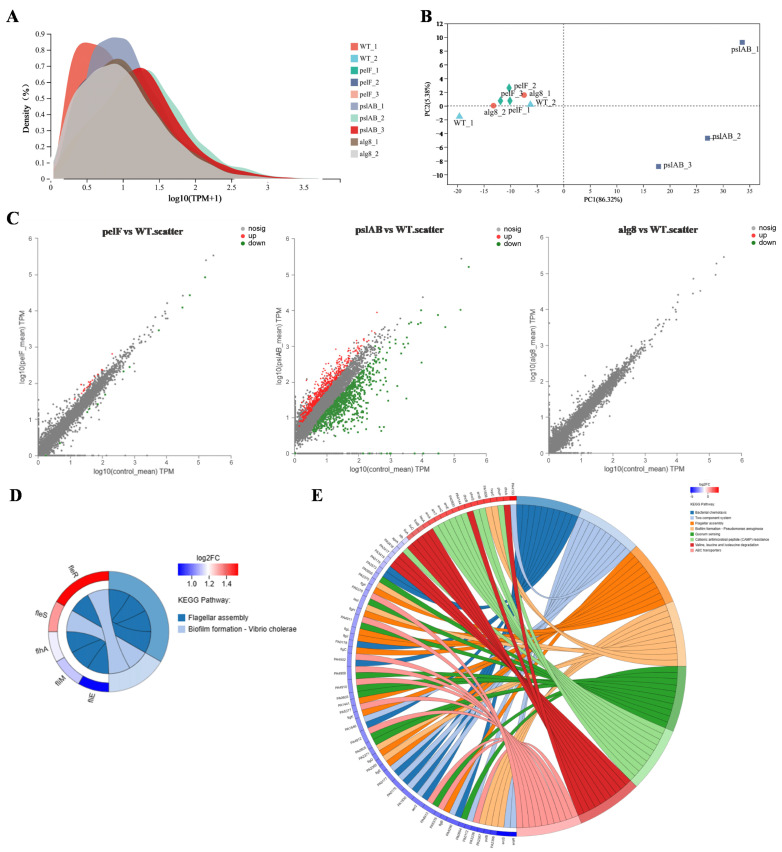
Transcriptomic analysis of biofilms. (**A**) Expression distribution density after removal of abnormal samples. (**B**) PCA after removal of abnormal samples. (**C**) Scatter plot of differential expressed genes among three comparison groups (PAO1-ΔpelF vs. PAO1-WT, PAO1-ΔpslAB vs. PAO1-WT, and PAO1-Δalg8 vs. PAO1-WT). (**D**) KEGG enrichment pathway of differential genes in PAO1-ΔpelF vs. PAO1-WT. (**E**) KEGG enrichment pathway of differential genes in PAO1-ΔpslAB vs. PAO1-WT.

To investigate the intrinsic regulatory mechanism of the core extracellular polysaccharide biosynthesis pathways in biofilm formation in *P. aeruginosa*, we employed transcriptomics to assess the transcription profiles of strains PAO1-WT, PAO1-Δ*pelF*, PAO1-Δ*pslAB*, and PAO1-Δ*alg8*. RNA quality inspection results indicated that the integrity of the RNA samples was satisfactory (Table S2). Transcriptome analysis of 12 samples confirmed that the sequencing data met the quality control standards (Table S3). The clean reads from each sample were aligned with strain PAO1 reference genome (Table S4), demonstrating high sequencing saturation and sufficient coverage of the majority of expressed genes. A total of 8464 expressed genes were identified, including 5532 known genes, 2779 novel genes, and 153 sRNA genes. Expression density distribution and principal component analysis revealed significant differences between samples PAO1-WT_3 and PAO1-Δalg8_3 compared to the others (Figure S1), likely due to batch effects. Consequently, these two samples were excluded from further analysis. The peak expression levels in the remaining ten samples were concentrated within the same region. Principal component analysis (PCA) showed distinct clustering of each sample group, with the PAO1-Δ*pslAB* group samples being notably more distant from the other three groups (Figure 2A,B), indicating that deletion of the *pslAB* gene significantly alters the gene expression profile of *P. aeruginosa* PAO1 biofilm formation.

Differential gene analysis, based on quantitative expression results, was performed to identify DEGs between groups (Figure 2C). The analysis revealed a total of 36 DEGs in strain PAO1-Δ*pelF* compared to strain PAO1-WT, with 24 upregulated and 12 downregulated. In contrast, 1293 DEGs were found between strain PAO1-Δ*pslAB* and PAO1-WT, with 501 upregulated and 792 downregulated. Notably, no DEGs were found in the comparison between strains PAO1-Δ*alg8* and PAO1-WT. KEGG functional enrichment analysis of the DEGs between strains PAO1-Δ*pelF* and PAO1-WT highlighted significant involvement in pathways such as flagellar assembly and biofilm formation (Figure 2D). Similarly, KEGG analysis of the DEGs between strains PAO1-Δ*pslAB* and PAO1-WT indicated enrichment in pathways associated with bacterial chemotaxis, two-component system, flagellar assembly, biofilm formation, quorum sensing (QS), cationic antimicrobial peptide (CAMP) resistance, valine, leucine and isoleucine degradation, and ABC transporters (Figure 2E).

**Figure 3:**
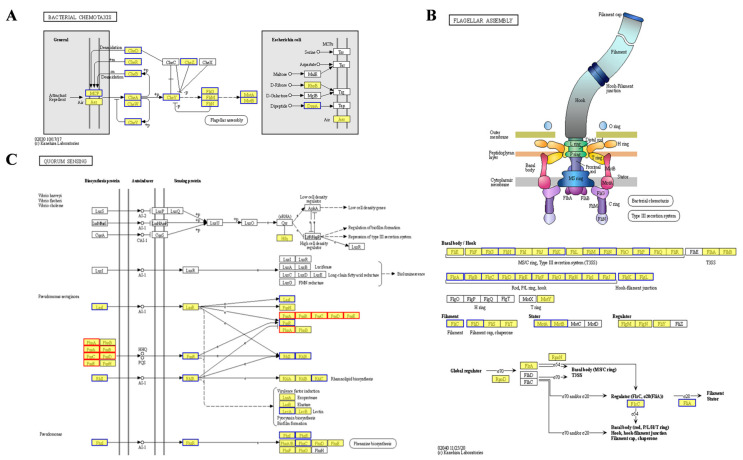
Differential KEGG pathway analysis of biofilms. (**A**) Bacterial chemotaxis in PAO1-Δ*pslAB* vsPAO1-WT. (**B**) Flagellar assembly in PAO1-ΔpslAB vs. PAO1-WT. (**C**) Quorum sensing in PAO1-Δ*pslAB* vs. PAO1-WT.

The two-component regulatory system FleS/FelR plays a pivotal role in governing the expression of a suite of genes central to flagellar synthesis, adhesion, aggregation, motility, and antibiotic resistance [20,21,22]. In strain PAO1-Δ*pelF*, compared to PAO1-WT, the upregulation of *fleS* and *felR* genes enhances flagellar synthesis efficiency in biofilms, thereby improving cell motility. Flagella is indispensable to the structural integrity of *P. aeruginosa* biofilm within genes such as *fliM*, *fliE*, and *flhA*, serving as key components in flagellar assembly [23]. Increased expression of these genes in strain PAO1-Δ*pelF* boosts cell motility, bacterial dispersion, and subsequent biofilm formation. Bacterial chemotaxis, the bacteria’s navigational response to chemical gradients in their surroundings, is fundamental to their quest for nutrients. This innate ability directs bacteria toward regions abundant in food molecules or away from noxious zones [24]. In contrast, strain PAO1-Δ*pslAB* shows a significant downregulation of genes associated with these functions. Specifically, 37 genes in the bacterial chemotaxis pathway were downregulated (Figure 3A), implicating their role in flagellar assembly [25]. Additionally, 24 genes within the flagellar assembly pathway were noted to be downregulated (Figure 3B), leading to reduced flagellar synthesis, diminished bacterial motility, and impaired biofilm formation.

Quorum sensing is a sophisticated bacterial communication mechanism essential for the regulation of collective behaviors, including EPS secretion, biofilm formation, and the adhesion and aggregation of bacterial communities [26]. In *P. aeruginosa*, four QS systems—the Las, Rhl, IPS, and PQS systems—form an elaborate QS regulatory network [27]. Comparative analysis of strain PAO1-Δ*pslAB* versus strain PAO1-WT reveals distinct expression patterns among QS-associated genes. Specifically, there is a downregulation of LasI and RhlI/RhlR, accompanied by an upregulation of *phnA* and *pqsABCDE* in the pqs system, which are essential for signal molecule synthesis (Figure 3C). This suggests a predominant influence of the pqs system within the QS network of strain PAO1-Δ*pslAB*. Additionally, the QS system’s regulatory impact extends to a suite of virulence factors, as evidenced by the diminished expression of genes such as lectin *lecA*, rhamnolipid synthesis gene *rhlC*, alkaline protease *aprA*, and its secreted protein *aprDF* (Figure 3C and Table S5). The ABC transporter pathway also exhibits heightened expression of MexGHI-OpmD, a multidrug efflux pump essential for modulating the growth and virulence of *P. aeruginosa*. This pump functions through 4-quinolone-mediated intercellular communication, regulated by the pqs system [28,29]. RT-qPCR was subsequently employed to validate the transcriptomic findings (Figure S2).

### 2.3. Metabolic Differences in P. aeruginosa Biofilm Mediated by the Core Extracellular Polysaccharide Biosynthesis Pathways

**Figure 4:**
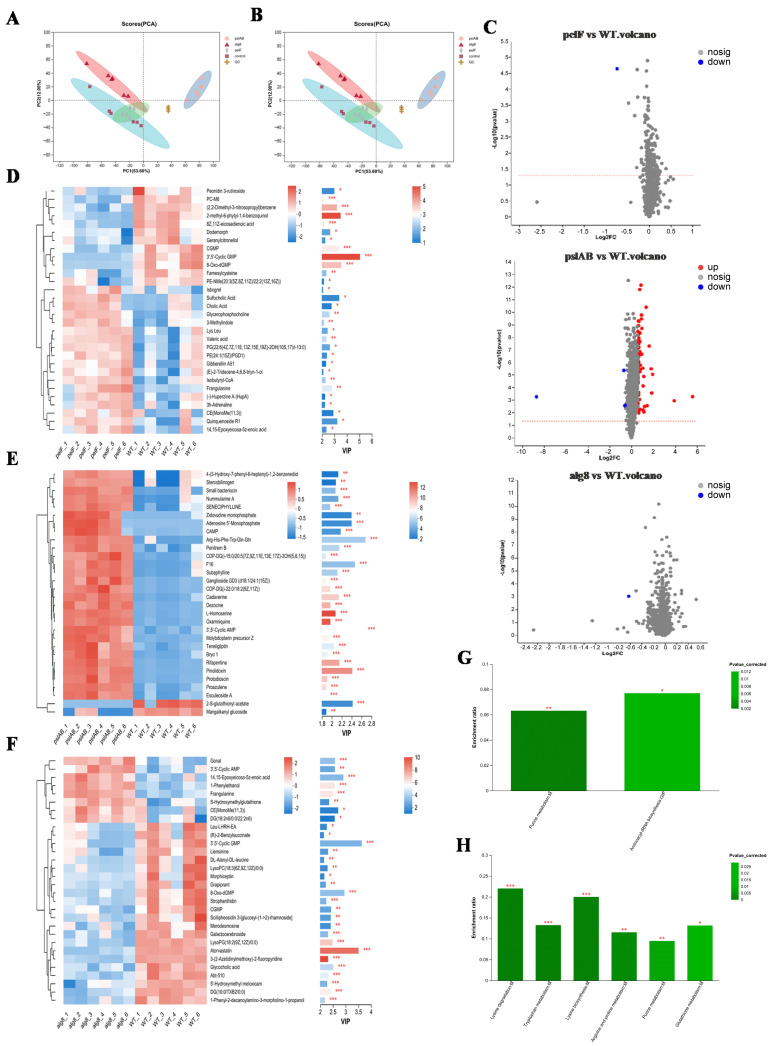
Metabolomic analysis of biofilms. (**A**) PCA scores of all samples in positive ion mode. (**B**) PCA scores of all samples in negative ion mode. (**C**) Volcano plot of significantly differential metabolites in the three comparison groups (PAO1-Δpelf Vs. PAO1-WT, PAO1-Δpslab Vs. PAO1-WT, and PAO1-Δalg8 Vs. PAO1-WT). (**D**) Cluster heatmap and VIP of metabolites in PAO1-Δpelf Vs. PAO1-WT. On the left is the clustering dendrogram of metabolites—branches that are closer indicate more similar expression patterns of all metabolites within the samples; On the right is the VIP bar plot, where the bar length (*, **, ***) represents the contribution value of each metabolite to group differences. A longer bar (or more asterisks) signifies a larger difference in that metabolite between the two groups. (**E**) Cluster heatmap and VIP of metabolites in PAO1-Δpslab Vs. PAO1-WT. (**F**) Cluster heatmap and VIP of metabolites in PAO1-Δalg8 Vs. PAO1-WT. (**G**) KEGG enrichment pathway of differential metabolites in PAO1-Δpelf Vs. PAO1-WT. (**H**) KEGG enrichment pathway of differential metabolites in PAO1-Δpslab Vs. PAO1-WT.

The total ion current (TIC) of the quality control (QC) samples (Figure S3) indicates that instrument-induced errors during the experimental process were minimal. PCA was utilized for metabolomics analysis to evaluate the discrepancies between different groups and within samples, with the results presented in Figure 4A,B. A clear distinction is observed between PAO1-Δ*pslAB* and PAO1-WT groups, with no overlap or intersection, effectively differentiating the two and indicating significant differences in metabolite profiles. Furthermore, the tight clustering of the QC samples in the figures signifies excellent system stability and repeatability. The R^2^x(cum) values (cumulative explanatory power) in both positive and negative ion modes are all greater than 0.5 (Table S6), indicating that the model has good fitting accuracy and is stable and reliable. The application of Partial Least Squares Discriminant Analysis (PLS-DA) and Orthogonal Partial Least Squares Discriminant Analysis (OPLS-DA) further confirms that the robustness and reliability of the model, along with its predictive capabilities (Figures S4–S7; Table S7 and Table S8). Comparative analysis revealed 75 significantly differential metabolites between PAO1-Δ*pslAB* and PAO1-WT groups, with 71 upregulated and 4 downregulated. In comparisons involving PAO1-Δ*pelF* and PAO1-Δ*alg8* groups versus PAO1-WT, a single significantly different metabolite, 3′,5′-Cyclic GMP (c-di-GMP), exhibited a downward trend (Figure 4C; Tables S9–S11). To demonstrate the expression patterns of metabolites across different groups within each sample and to visually discern the significance and trend changes in differential metabolites, cluster analysis and Variable Importance in Projection (VIP) value analysis were conducted on differential metabolites (Figure 4D–F). The results showed that each knockout group, as well as the wild-type group, exhibits unique metabolite expression patterns, especially between PAO1-Δ*pslAB* and PAO1-WT groups. Notably, most metabolites in the PAO1-Δ*pslAB* group were found to be accumulated, with only a minor portion consumed.

KEGG functional enrichment analysis was conducted on the differential metabolites among various groups. In the comparison between PAO1-Δ*pelF* and PAO1-WT groups, the differential metabolites were primarily associated with Purine metabolism and Aminoacyl-tRNA biosynthesis pathways (Figure 4G). In contrast, differential metabolites between the PAO1-Δ*pslAB* and PAO1-WT groups were predominantly involved in Lysine degradation, Tryptophan metabolism, Lysine biosynthesis, Arginine and proline metabolism, Purine metabolism, and Glutathione metabolism. The PAO1-Δ*alg8* group, when compared to the PAO1-WT group, did not exhibit any significantly enriched metabolic pathways (Figure 4H). The significantly different metabolite between PAO1-Δ*pelF* and PAO1-WT groups notably characterized by a diminished c-di-GMP level (Table S9). Concurrently, a KEGG pathway enrichment analysis of the differential metabolites revealed that the purine metabolism pathway was significantly enriched in the comparison between PAO1-Δ*pelF* and PAO1-WT groups. As illustrated in Figure S8, this enrichment included a total of four upregulated and two downregulated metabolites. This observation is attributed to the degradation of c-di-GMP into GMP, which subsequently breaks down, leading to an accumulation of degradation products including hypoxanthine, xanthine, uric acid, and adenosine. In comparison between PAO1-Δ*pslAB* and PAO1-WT groups, metabolic pathways associated with Lysine (Lys), Arginine (Arg), Proline (Pro), and Tryptophan (Trp) were found to be significantly enriched with nearly all metabolites upregulated (Table S12). This upregulation suggests that these amino acids were predominantly in a state of catabolism. The observed upregulation may be associated with the diminished biofilm formation in strain PAO1-Δ*pslAB*, where extracellular proteins were not secreted extracellularly in large quantities and are consequently degraded into amino acids and their metabolic byproducts.

### 2.4. Combined Transcription-Metabolism Analysis of P. aeruginosa Biofilm Mediated by Core Extracellular Polysaccharide Biosynthesis Pathways

#### 2.4.1. The Psl Extracellular Polysaccharide Biosynthesis Pathway Affects the Polysaccharide Metabolism of Biofilms

**Figure 5:**
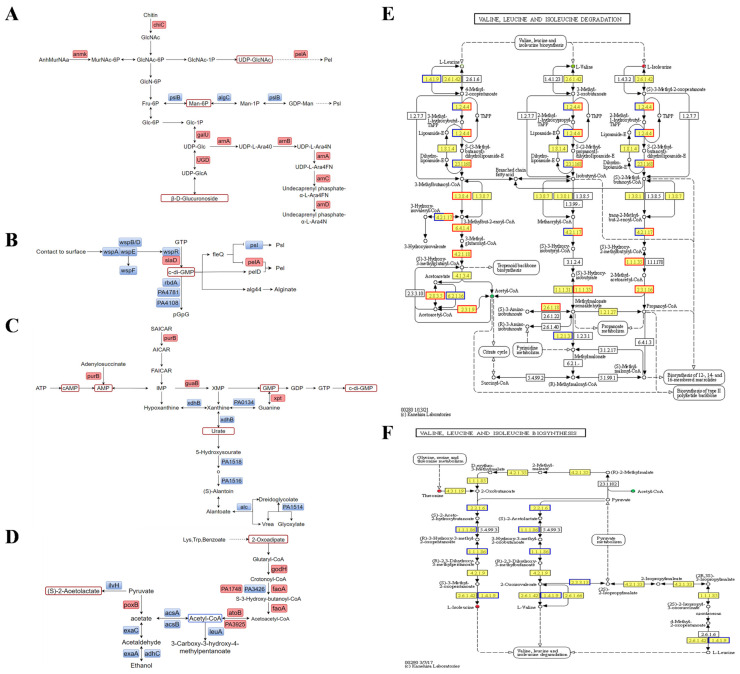
Combined transcription-metabolism analysis of biofilms. (**A**) Polysaccharide metabolism in PAO1-Δpslab Vs. PAO1-WT. (**B**) Regulation of c-di-GMP in PAO1-Δ*pslAB* vs. PAO1-WT. (**C**) C-di-GMP biosynthesis in PAO1-Δ*pslAB* vs. PAO1-WT. (**D**) Acetyl-CoA metabolism in PAO1-Δ*pslAB* vs. PAO1-WT. (**E**) Valine, leucine, and isoleucine degradation in PAO1-Δ*pslAB* vs. PAO1-WT. (**F**) Valine, leucine, and isoleucine biosynthesis in PAO1-Δ*pslAB* vs. PAO1-WT.

To comprehensively and systematically elucidate the formation mechanism of *P. aeruginosa* biofilm mediated by the core extracellular polysaccharide biosynthetic system, differential genes and metabolites between PAO1-Δ*pslAB* and PAO1-WT groups were co-enriched in the pathway. Integrated analysis of transcriptional and metabolic data revealed that the knockout of *pslAB* directly impacted the synthesis of GDP-Man, culminating in the intracellular accumulation of Man-6P (Figure 5A). In the Psl extracellular polysaccharide biosynthetic system, PslB is responsible for a two-step catalytic reaction. Firstly, it converts Fru-6P to Man-6P, and after converting Man-6P to Man-1-phosphate in AlgC, PslB further catalyzes the synthesis of GDP-mannose (GDP-Man), which is one of the key sugar nucleotide precursors for of Psl polysaccharide biosynthesis [7]. The absence of GDP-Man impedes the synthesis of *P. aeruginosa* PAO1’s main extracellular polysaccharide, Psl.

The concentration of UDP-N-acetylglucosamine (UDP-GlcNAc) significantly increased in strain PAO1-Δ*pslAB* compared to PAO1-WT, reflecting the bacterium’s dual approach to enhance GlcNAc synthesis. This strategy includes the upregulation of *chiC* expression on one hand, and the enhancement of *anmK* expression on the other. Moreover, the expression of *pelA* has witnessed an uptick. Recent research has identified that Pel polysaccharide comprises repeating units of N-acetylglucosamine (GalNAc) and galactosamine (GalN) [30]. In the Pel biosynthesis pathway, PelX functions as a UDP-GlcNAc C4 epimerase, converting UDP-GlcNAc to UDP-GalNAc [31]. Concurrently, PelA, which has periplasmic chaperone and deacetylase activities, is responsible for partially deacetylating the Pel polysaccharide [32]. The elevated of UDP-GlcNAc levels in strain PAO1-Δ*pslAB* likely serve as a precursor for the Pel polysaccharide synthesis, with UDP-GalNAc as the sugar-nucleotide progenitor. Moreover, the increased expression of *pelA* suggests enhanced Pel polysaccharide production following the inability to synthesize Psl in strain PAO1-Δ*pslAB*. The absence of *pslAB* genes halts the conversion of Fru-6P to Man-6P, prompting PAO1-Δ*pslAB* to upregulate *galU* and *PA3559*, resulting in increased β-D-Glucuronoside levels. Additionally, UDP-Glc produced by *galU* is converted into 4-amino-4-deoxy-1-arabinose (L-AraN) under the action of *arnBCADTEF* enzyme complex (Figure 5A). The expression of the *arn* operon was upregulated by 3–4.7 times, with ArnT facilitating the transfer of the positively charged amino arabinose unit to the 4-phosphate position of lipid A, thereby reducing the electronegativity of the lipopolysaccharide and enhancing resistance to cationic antimicrobial peptides [33]. The upregulation of the *arn* operon is regulated by the two-component system PhoP/PhoQ, activated under acidic pH or low Mg^2+^/Ca^2+^ conditions (Table S5).

#### 2.4.2. The Absence of Psl Extracellular Polysaccharide Biosynthesis Pathway Enhances the Synthesis of C-di-GMP

C-di-GMP plays a central role in regulating various aspects of bacterial collective behavior, including biofilm formation in *P. aeruginosa* [34]. The metabolism of c-di-GMP is regulated by two key enzymes: diguanylate cyclase (DGC), which synthesizes c-di-GMP from two GTP molecules, and phosphodiesterase (PDE), which degrades c-di-GMP into pGpG. Within bacteria, a range of DGCs and PDEs are present, as depicted in Figure 5B. Specifically, the DGCs *wspR* and *siaD*, along with the PDEs *rbdA*, *PA4781*, and *PA4108*, exhibit differential expression patterns between PAO1-Δ*pslAB* and PAO1-WT groups. The downregulation of all three PDEs anticipated to elevate c-di-GMP level. Conversely, the expression of DGCs follows varied trajectories: the upregulation of *siaD* contributes to increased c-di-GMP, whereas *wspR* shows a decreasing trend in expression. The interaction among these enzymes results in a higher concentration of c-di-GMP. Given the capacity of c-di-GMP to bind with FleQ, thereby eliminating the inhibitory effect of FleQ on the *pel* operon [35], the augmented levels of c-di-GMP are likely to intensify its interaction with *fleQ*, abate the repression of the *pel* operon, and subsequently upregulate the expression of *pelA*.

The expression level of WspABDEFR, a key component in c-di-GMP regulation, was found to be decreased by 2-fold in the PAO1-Δ*pslAB* group compared to the PAO1-WT group (Figure 5B). The Wsp system operates as a surface-sensing mechanism akin to cellular tropism, playing a key role in the detection of mechanical forces at the bacterial surface. It regulates the biofilm formation and extracellular polysaccharides by regulating c-di-GMP levels [36]. The observed downregulation of Wsp hinders that strain PAO1-Δ*pslAB* has diminished surface adhesion, which may hinder biofilm formation.

The synthesis pathway of c-di-GMP in strain PAO1-Δ*pslAB* is shown in Figure 5C. Within the bacterial cell, elevated levels of cAMP and AMP contribute to GMP production through the purine metabolism, culminating in the synthesis of c-di-GMP. The upregulation of *purB* and *guaB* genes facilitates this biosynthetic process. Concurrently, the downregulation of six genes associated with purine degradation indicates a preferential allocation of nucleotides towards the synthetic pathway rather than the catabolic pathway.

Metabolomics studies reveal a significant reduction in acetyl-CoA levels in strain PAO1-Δ*pslAB*. Integrated transcriptional and metabolic analyses indicate that multiple acetyl-CoA-related pathways are regulated. Notably, genes *gcdH*, *faoA*, and *atoB*, involved in the Trp, Lys, and benzoic acid metabolic pathways, respectively, are consistently upregulated, which is essential for acetyl-CoA synthesis (Figure 5D). Conversely, six genes in the synthesis pathway of the Val, Leu, and Ile were found to be continuously downregulated, while the differentially expressed genes in their degradation pathway were predominantly upregulated. This suggests a shift in these amino acids towards acetyl-CoA synthesis (Figure 5E,F). Additionally, in pyruvate metabolism, alcohol metabolism is inhibited, and the expression of multiple genes is downregulated. Notably, the expression of *acsAB* has decreased by 4–7-fold, and that of *exaAC* has decreased by 16–18-fold (Figure 5D). This suggests a decline in anaerobic respiration and an increased reliance on aerobic respiration for acetyl-CoA synthesis. Despite the regulation of various pathways for acetyl-CoA production, its overall content is reduced, likely due to its utilization in the TCA cycle (tricarboxylic acid cycle) for ATP production, which subsequently increases c-di-GMP levels.

#### 2.4.3. The Absence of Psl Extracellular Polysaccharide Biosynthesis Pathway Alters the Iron Ion Uptake Mode of Biofilm

Iron ions are essential for the normal physiological metabolism of bacteria, significantly influencing intercellular communication and biofilm formation [37]. The acquisition of iron is a critical determinant of the infectious capacity of *P. aeruginosa* [38]. *P. aeruginosa* acquires iron through the production of high-affinity iron-chelating siderophores, including pyochelin, pyoverdine, the phenazine derivative pyocyanine, and enterobactin [38]. In strain PAO1-Δ*pslAB*, we observed a 2–5-fold downregulation of the synthesis gene clusters *pchABCDEFG* (for pyochelin) and *pvdADEFGHJL* (for pyoverdine), as well as significant downregulation of their respective receptors, *fpvB* and *fptA*. In contrast, the gene *phzHS*, involved in pyocyanine synthesis, and the regulatory transport genes *pfeASR* and the periplasmic binding protein *fepB*, associated with enterobactin, were upregulated by 2–3-fold (Table S13). Additionally, the pyocyanine content increased. Although the VIP value for pyocyanine is less than 1, it suggests that the loss of function in the Psl biosynthesis pathway has shifted the iron chelation strategy of *P. aeruginosa*, leading to primary reliance on pyocyanine and enterobactin for iron acquisition to sustain normal bacterial growth.

## 3. Discussion

This article provides new insight into the mechanism of *P. aeruginosa* biofilm formation, specifically mediated by three core extracellular polysaccharide biosynthesis pathways. It represents the most comprehensive study on core extracellular polysaccharide biosynthesis pathways of *P. aeruginosa* to date. Initially, biofilms in mutant strains PAO1-Δ*pelF*, PAO1-Δ*pslAB*, and PAO1-Δ*alg8* were analyzed qualitatively and quantitatively using crystal violet staining, SEM observation, CLSM measurement, and QCM-D real-time monitoring. Subsequently, a combined transcriptional and metabolic omics approach was employed to investigate the internal regulatory mechanism *P. aeruginosa* biofilm formation. This analysis aimed to systematically elucidate the roles of three extracellular polysaccharide biosynthesis pathways in biofilm formation and to clarify the dynamic mechanisms of biofilm formation mediated by extracellular polysaccharide biosynthetic systems from phenotypic, transcriptional, and metabolic perspectives. The findings indicate that the Psl biosynthesis pathway plays a key role in the formation of *P. aeruginosa* biofilm. The Pel biosynthesis pathway has a certain effect on the formation of *P. aeruginosa* biofilm. The alginate biosynthesis pathway has the least impact on effect on the formation of *P. aeruginosa* biofilm.

The Psl biosynthesis pathway plays a key role in the formation of *P. aeruginosa* biofilm. Various analytical techniques used in this study revealed that strain PAO1-Δ*pslAB* exhibits reduced biofilm formation and thickness. Real-time monitoring via QCM-D showed no significant increase in bacterial adherence and proliferation during the growth phase. These findings underscore Psl’s critical role in *P. aeruginosa* biofilm formation; in the absence of Psl synthesis, biofilm formation is severely impaired [39]. The reduced biofilm formation in PAO1-*ΔpslAB* also resulted in a doubling of biofilm roughness compared to strain PAO1-WT. QCM-D analysis further revealed that the elastic modulus of PAO1-*ΔpslAB* biofilm was significantly increased compared to PAO1-WT. Research indicates that the Psl biosynthesis pathway promotes the persistence of cells under high-shear flow conditions by providing strong adhesive properties [9]. Chew et al. demonstrated that Psl improves biofilm matrix cross-linking and elasticity, strengthens the scaffold structure, and aids in microcolony formation [10]. Consistent with these findings, the absence of functional Psl results in a marked increase in elastic modulus and a decrease in biofilm elasticity. Differential gene expression analysis revealed a six-fold reduction in rsaL expression in PAO1-*ΔpslAB* compared to PAO1-WT (Table S5). RsaL is a global gene regulator in *P. aeruginosa* involved in quorum sensing homeostasis [40], with lasR inducing RsaL expression, which then inhibits pyocyanine synthesis by binding to its promoter [41]. Consequently, the downregulation of *lasR* in strain PAO1-Δ*pslAB* resulted in decreased expression of *rsaL*, relieving the inhibition on pyocyanine synthesis and upregulating the expression of *phzH* and *phzS*, thus increasing pyocyanine levels. Additionally, gene expression related to alginate synthesis, such as *algB* and *mucBCD*, showed an upregulation trend in strain PAO1-Δ*pslAB* (Table S5) [42]. Wiens et al. found that the loss of pyoverdine eliminated the ability of *P. aeruginosa* to produce Psl-type biofilms. Mutations in genes involved in pyoverdine and pyochelin biosynthesis, as well as the pyoverdine receptor *fpvA*, resulted in the iron-dependent constitutive expression of the alginate biosynthesis gene *algD*, culminating in a distinct mucinous phenotype [42]. This may explain the slightly higher viscosity (albeit not significant) observed in strain PAO1-Δ*pslAB*, as indicated by the epigenetic measurement results: the downregulation of pyoverdine, pyochelin, and other related genes in strain PAO1-Δ*pslAB* increases the expression of *mucBCD*, enhances alginate synthesis, and consequently makes the biofilm more viscous, thereby potentially increasing the viscosity.

The experiment conducted by An et al. demonstrated that the deletion of *rbdA* in *P. aeruginosa* PAO1 led to an increase in extracellular polymeric substance (EPS) production, predominantly through the synthesis of Pel polysaccharides [43]. Transcriptional analysis of the *pelA* gene in the Δ*rbdA* mutant revealed that the expression level of *pelA* was elevated by 1.5-fold compared to strain PAO1-WT. Additionally, the binding of c-di-GMP to FleQ can counteract the inhibitory effect of FleQ on the *pel* operon [35]. This observation aligns with the results of this study, suggesting that the downregulation of *rbdA* increases c-di-GMP levels. The elevated c-di-GMP then binds to *fleQ*, thereby alleviating the inhibition of *pelA* and consequently upregulates its expression. Consequently, these findings imply that strain PAO1-Δ*pslAB* depends on Pel polysaccharides to form biofilms. However, due to reduced flagellar synthesis and decreased expression of colonization factors in PAO1-*ΔpslAB*, the strain exhibits significantly impaired motility and forms only a sparse biofilm, failing to achieve normal biofilm levels.

The Pel biosynthesis pathway has a certain effect on the formation of *P. aeruginosa* biofilm. Epigenetic assay indicated that the biofilm biomass in strain PAO1-Δ*pelF* experienced only a modest reduction, consistent with the findings reported by Colvin et al. [39]. This observation suggests that the Pel biosynthesis pathway functions as a structural scaffold in mature biofilms, providing a redundant role. Metabolomics analysis revealed analysis c-di-GMP levels in strain PAO1-Δ*pelF*, while transcriptomics data demonstrated the upregulation of genes associated with flagellar assembly. Prior research has demonstrated that flagellar genes are more highly expressed at low c-di-GMP concentrations [35]. Thus, the Pel biosynthesis pathway appears to negatively regulate flagellar synthesis and bacterial motility by modulating c-di-GMP levels within the biofilm, thereby influencing biofilm formation. Nonetheless, this regulatory mechanism does not fully compensate for the functional deficits associated with the absence of the Pel biosynthesis pathway in the *P. aeruginosa*.

The alginate extracellular polysaccharide biosynthesis pathway has the least impact on the effect on the formation of *P. aeruginosa* biofilm. Analyses of metabolomics and transcriptomics data revealed no substantial differences in physiological processes, indicating that the alginate extracellular polysaccharide biosynthetic system neither significantly promotes nor inhibits biofilm formation in *P. aeruginosa* PAO1 under laboratory conditions. Current research has not demonstrated a role for alginate in biofilm formation of *P. aeruginosa* in vitro models utilizing glass or plastic as substrates [34]. These findings suggest that the alginate biosynthesis pathway has the least impact on effect on the formation of PAO1 biofilm. Alipour et al. mentioned that exogenous alginate lyase (AlgL) may improve the availability of drugs to *P. aeruginosa* from the sputum of patients with cystic fibrosis (CF) [44]. This apparent discrepancy may arise from contextual differences. This suggests that, although alginate may not directly contribute to biofilm structural assembly, it could still play a pivotal role in bacterial pathogenicity and antibiotic accessibility. Such a discovery establishes a mechanistic rationale for developing enhanced therapeutic strategies targeting CF.

## 4. Materials and Methods

### 4.1. Strains Cultivation

The *P. aeruginosa* strains PAO1-WT, PAO1-Δ*pelF*, PAO1-Δ*pslAB*, and PAO1-Δ*alg8* used in this study were preserved strains in our laboratory. Strains stored in −80 °C glycerol tube were placed on LB agar plates (Beijing Land Bridge Technology Co., Ltd., Beijing, China) containing 30 µg/mL kanamycin (Sangon Biotech Co., Ltd., Shanghai, China) crossed line, cultured at 37 °C for 18–24 h. Single colonies were selected and inoculated in LB broth (Beijing Land Bridge Technology Co., Ltd., Beijing, China) containing 30 µg/mL kanamycin and shaken at 37 °C for 18 h.

### 4.2. Biofilm Assays

#### 4.2.1. Crystal Violet Staining

Referring to the previous method in our laboratory [45], in brief, 100 µL of 6 Log CFU/mL bacterial solution was added to a 96-well plate and incubated for 24 h at 25 °C. Then, the plate was rinsed with PBS buffer (Sangon Biotech Co., Ltd., Shanghai, China). Next, 150 µL of 0.1% crystal violet (Sangon Biotech Co., Ltd., Shanghai, China) was added for staining, followed by careful washing away of unbound crystal violet with PBS buffer. Finally, 200 µL of 95% ethanol (Sinopharm Chemical Reagent Co., Ltd., Shanghai, China) was added. The absorbance was measured at 595 nm.

#### 4.2.2. Scanning Electron Microscope (SEM)

Referring to the previous method in our laboratory [46]. In brief, biofilm was cultivated in a 24-well plate (Corning Management Co., Ltd., Shanghai, China), fixed with 4% glutaraldehyde (Shanghai Yuanye Bio-Technology Co., Ltd., Shanghai, China) and 30%, 50%, 70%, 90%, and 100% ethanol for 10 min each, and finally dehydrated with 100% ethanol for another 10 min. The samples were observed using an Extreme-resolution Analytical Field Emission scanning electron microscope (SEM) (Tescan Mira 3 MH, Brno, Czechia).

#### 4.2.3. Confocal Laser Scanning Microscopy (CLSM)

Referring to the previous methods in our laboratory [47], in brief, the biofilms were stained with 1 mL SYBR Green I dye (Beijing Solarbio Science & Technology Co., Ltd., Beijing, China) per well at room temperature for 30 min in the dark and rinsed again. The cover glass was placed on the slide and then observed using Leica TCS SP8 (Leica Microsystems, Wetzlar, German).

#### 4.2.4. Quartz Crystal Microbalance with Dissipation (QCM-D)

Following the method of Olsson et al. [48], in brief, QCM-D silicon dioxide chips were sequentially cleaned and overnight soaking in 2% SDS (Sangon Biotech Co., Ltd., Shanghai, China) and 2% HellmanexTM (Sigma-Aldrich Co., Ltd., Shanghai, China). Then, they were irradiated with ultraviolet light for 20 min before being installed in the QCM-D (Biolin Scientific, Gothenburg, Sweden) chamber. The control experiment, conducted using sterile LB, confirmed that there was no contamination in the QCM-D system (i.e., no membrane growth was detected).

### 4.3. Biofilm Sample Preparation

Referring to the method of Tian et al. [47], mutant strains of *P. aeruginosa* were constructed, including PAO1-Δ*pelF*, PAO1-Δ*pslAB*, and PAO1-Δ*alg8*. Biofilm was cultivated in a 90 mm diameter Petri dish and washed with 10 mL of PBS buffer. Biofilm was scraped from the bottom of the dish to a 1.5 mL sterile centrifuge tube. The samples were frozen in liquid nitrogen and stored at −80 °C. For transcriptomic analysis, a total of 4 groups were prepared, each with 3 replicates, named as follows: PAO1-WT group, PAO1-Δ*pelF* group, PAO1-Δ*pslAB* group, and PAO1-Δ*alg8* group. The collected samples were delivered to Shanghai Meiji Biomedical Technology Co., Ltd., Shanghai, China, for multi-omics analysis.

### 4.4. Transcriptomics

#### 4.4.1. RNA Extraction and Library Construction

The total bacterial RNA was extracted using the TRIzol^®^ reagent (Invitrogen, Waltham, MA, USA) according to the manufacturer’s instructions, genomic DNA was removed using DNase I (Invitrogen, Waltham, MA, USA), RNA quality was initially determined by agarose gel electrophoresis, and RNA integrity (RIN value) was determined using an Agilent 2100 bioanalyzer (Agilent, Santa Clara, CA, USA). RNA was quantified using an ND-2000 (NanoDrop Technologies, Wilmington, DE, USA). High-quality RNA samples were used for subsequent library construction. RNA extraction and follow-up work were completed by Shanghai Meiji Biomedical Technology Co., Ltd., Shanghai, China. After quantification with TBS380, RNA-seq paired-end sequencing was performed using Illumina HiSeq X Ten (2 × 150 bp) (Illumina, San Diego, CA, USA).

#### 4.4.2. Sequencing Data Quality Control

The Illumina HiSeq X Ten converts sequencing image signals into text signals through CASAVA base calling and stores them in the fastq format as raw data. Statistical methods were employed to analyze base distribution and quality score fluctuations. Subsequently, each sample was evaluated for base quality, base error rate, and base composition distribution. In total, 10,000 raw reads were randomly selected from each sample using the blast method and compared them with the Rfam database 14.6. Based on the annotation results, the percentage of rRNA in each sample was calculated to evaluate the rRNA contamination rate.

#### 4.4.3. Sequence Comparative Analysis

The raw data after quality control were aligned with the PAO1 reference genome using the Burrows–Wheeler method to obtain mapped data. At the same time, the quality of the alignment results from this transcriptome sequencing was assessed, which mainly included sequencing saturation, gene coverage, the distribution of reads in different regions of the reference genome, and analysis of reads distribution across different chromosomes.

#### 4.4.4. Gene Expression Level Analysis

The software RSEM 1.3.1 was used to perform quantitative analysis of gene expression levels, with TPM (Transcripts Per Million) as the quantitative metric. PCA was conducted on the samples based on the expression profiles of genes/transcripts across different samples.

#### 4.4.5. Differential Analysis of Gene Expression Levels

We utilized the DESeq2 1.24.0 software to perform statistical analysis on raw counts and obtained significantly differentially expressed genes (DEGs) between comparison groups based on the screening criteria of an adjusted *p*-value (*p* adjust) < 0.05 and |log2FC| ≥ 1.

#### 4.4.6. Enrichment Analysis of Differentially Expressed Gene Functions

Utilizing an R script to perform KEGG PATHWAY enrichment analysis on differentially expressed genes, and employed the Benjamini–Hochberg (BH) method to adjust the *p*-value. When the *p*-adjust < 0.05, it is considered that there is significant enrichment in the KEGG pathway.

#### 4.4.7. RT-qPCR Validation

Based on the RNA-seq results, significantly differentially expressed genes *gcdH* and *fliM*, as well as the non-significantly differentially expressed gene *glcB*, were selected for RT-qPCR validation. 16S rRNA was chosen as the internal reference gene. Reverse transcription was performed using the HiScript Q RT SuperMix for qPCR (+gDNA wiper) kit (Nanjing Novozymes Biotechnology Co., Ltd., Nanjing, China), and fluorescence quantitative PCR was conducted on the obtained cDNA using the ChamQ SYBR Color qPCR Master Mix (2X) kit (Nanjing Novozymes Biotechnology Co., Ltd., Nanjing, China). Each sample was repeated three times, and the measurement results were calculated based on Ct (cycle threshold) values to determine ΔCt (difference in cycle threshold) value, ΔΔCt (difference in difference in cycle threshold) value, and 2^−ΔΔCt^ value. The relative expression level of the target gene was calculated based on the 2^−ΔΔCt^ value (Figure S2).

### 4.5. Metabolomics

#### 4.5.1. Sample Preparation

Take a sample of biofilm cells in a 2 mL centrifuge tube and add a grinding bead with a diameter of 6 mm. Extract metabolites using 400 µL of extraction solution (methanol: water = 4:1, *v/v*) containing 0.02 mg/mL of the internal standard (L-2-chloroalanine). The sample solution was ground in a frozen tissue grinder for 6 min at −10 °C and 50 Hz and then subjected to low-temperature ultrasonic extraction for 30 min at −5 °C and 40 kHz. Place the sample at −20 °C for 30 min, centrifuge at 13,000× *g* at 4 °C for 15 min, and transfer the supernatant to an injection vial with an insert tube for analysis. In addition, 20 µL of all sample’s metabolites were mixed to prepare a QC. During the instrument analysis process, 1 QC sample was inserted among every 5–15 analyzed samples to assess the repeatability and stability of the entire detection process.

#### 4.5.2. Liquid Chromatography-Tandem Mass Spectrometry (CL-MS/MS)

The instrument platform for LC-MS analysis is the ultra-high performance liquid chromatography tandem Fourier transform mass spectrometry (UHPLC-Q Exactive HF-X system, Thermo Fisher Scientific, Waltham, MA, USA). A 2 µL of sample was separated using an HSS T3 chromatography column (100 mm × 2.1 mm i.d., 1.8 µm). The injection volume was 3 µL, and the column temperature was set to 40 °C. The sample was ionized by electrospray, and mass spectrum signals were collected using both positive and negative ion scanning modes. The ion transfer tube temperature was maintained at 325 °C, and the normalized collision was a cyclic profile of 20–40–60 V. Data were acquired using the Data-Dependent Acquisition (DDA) mode.

#### 4.5.3. Data Processing

The LC-MS raw data were imported into the metabolomics processing software Progenesis QI (Waters Co., Ltd., Milford, CT, USA) for baseline filtering, peak identification, integration, retention time correction, peak alignment, and other processes, ultimately resulting in a data matrix that includes retention time, mass-to-charge ratio (*m/z*), and peak intensity. Then, the software was used for feature peak library identification.

After the database search was conducted, the matrix data were subjected to data preprocessing. The data matrix employed the 80% rule for eliminating missing values. The 80% rule was applied to remove missing values from the data matrix, followed by imputation of the remaining missing values. The sum normalization method was then used to normalize the response intensity of mass spectral peaks across samples, resulting in a normalized data matrix. Simultaneously, variables with a relative standard deviation (RSD) >30% in the QC samples were removed, and a log10 transformation was performed to obtain the final data matrix for subsequent analysis.

#### 4.5.4. Multivariate Statistical Analysis

Perform differential analysis on the preprocessed matrix file. The R software package 1.6.2 ‘ropls’ was utilized for PCA, PLS-DA, and OPLS-DA, with the model stability was evaluated through 7 cycles of interactive validation. Additionally, conduct Student’s *t*-tests and multiple comparison analysis.

#### 4.5.5. Differential Metabolite Analysis

The selection of differential metabolites was based on the Variable Importance for the Projection (VIP) values obtained from the OPLS-DA model and the *p*-values from Student’s *t*-tests. Metabolites with VIP > 1 and *p* < 0.05 are considered differential metabolites. These differential metabolites were annotated for metabolic pathways using the KEGG (Kyoto Encyclopedia of Genes and Genomes) database. Pathway enrichment analysis was conducted using the Python 3.7.7 package, and the most relevant biological pathways for the experimental treatment were obtained using Fisher’s exact test.

### 4.6. Statistical Analysis

Each experiment was independently repeated three times, and a single-factor analysis was performed on the experimental data using GraphPad Prism 9 software. CLSM images were analyzed using ISA-2, which was provided by Professor Haluk Beyenal of Montana State University. QCM-D data were analyzed using QSense Dind software v1.0.0. A test level of *p* < 0.05 was considered significant, and *p* < 0.01 was considered highly significant.

## 5. Conclusions

This study meticulously elucidates the roles of three extracellular polysaccharide biosynthesis pathways in the biofilm formation of *P. aeruginosa*: the Pel extracellular polysaccharide biosynthesis pathway has a certain effect on the formation of *P. aeruginosa* biofilm; the Psl extracellular polysaccharide biosynthesis pathway plays a crucial role in the formation of *P. aeruginosa* biofilm; and the alginate extracellular polysaccharide biosynthesis pathway has the least impact on effect on the formation of *P. aeruginosa* biofilm. Understanding these mechanisms is essential for advancing our knowledge of the resistance mechanisms inherent in *P. aeruginosa* biofilm. Such insights are important for developing and implementing effective preventative and control measures, thus enabling more effective management of the challenges posed by *P. aeruginosa* biofilms to environmental safety and public health.

## Data Availability

Data are contained within the article.

## Supplementary Materials

The following supporting information can be downloaded at: https://www.mdpi.com/article/10.3390/ijms26083780/s1.
