# Supplementary material for: New Insights into the Mediation of Biofilm Formation by Three Core Extracellular Polysaccharide Biosynthesis Pathways in Pseudomonas aeruginosa
Source: Int J Mol Sci. 2025 Apr 17;26(8):3780. doi: 10.3390/ijms26083780 (PMC12027665; doi:10.3390/ijms26083780)
Supplement: Supplementary file 1 [file ijms-26-03780-s001.zip › ijms-3546241-supplementary.pdf]

Supplementary materials

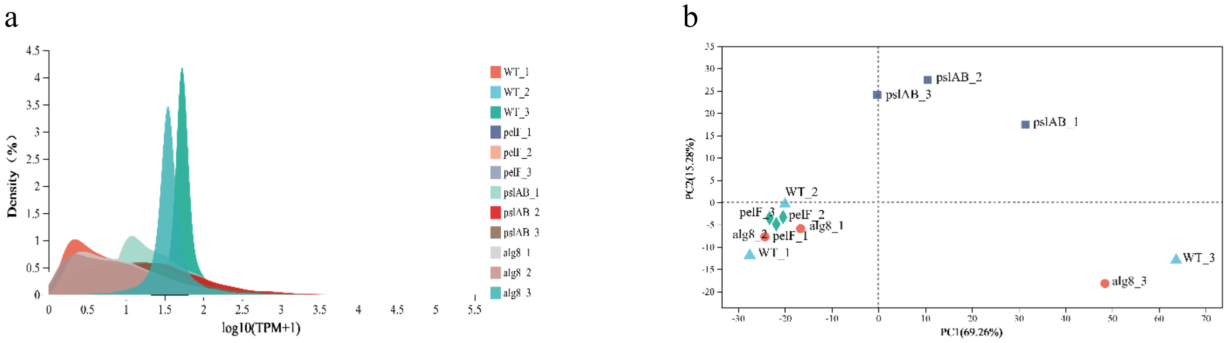

Figure S1. Analysis of gene expression. (a) Expression distribution density of 12 samples. (b) 1PCA analysis after removal of abnormal samples.

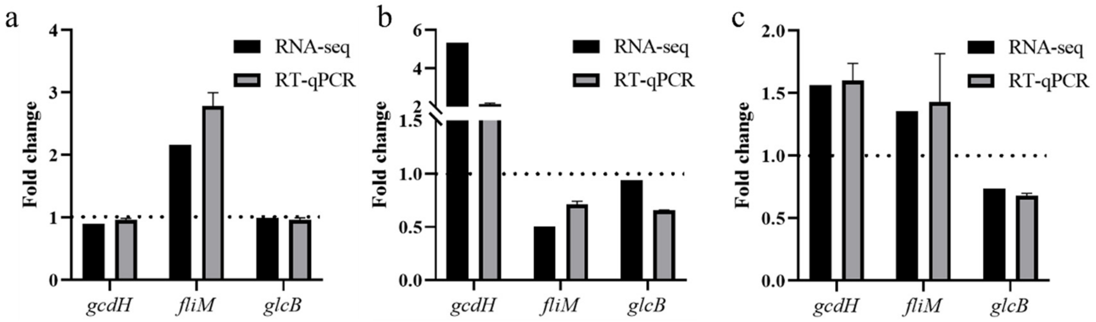

Figure S2. RT-qPCR validation of transcriptome sequencing. (a) PAO1-Δ*pelF* vs PAO1-WT. (b) PAO1-Δ*pslAB* vs PAO1-WT. (c) PAO1-Δ*alg8* vs PAO1-WT.

a

b

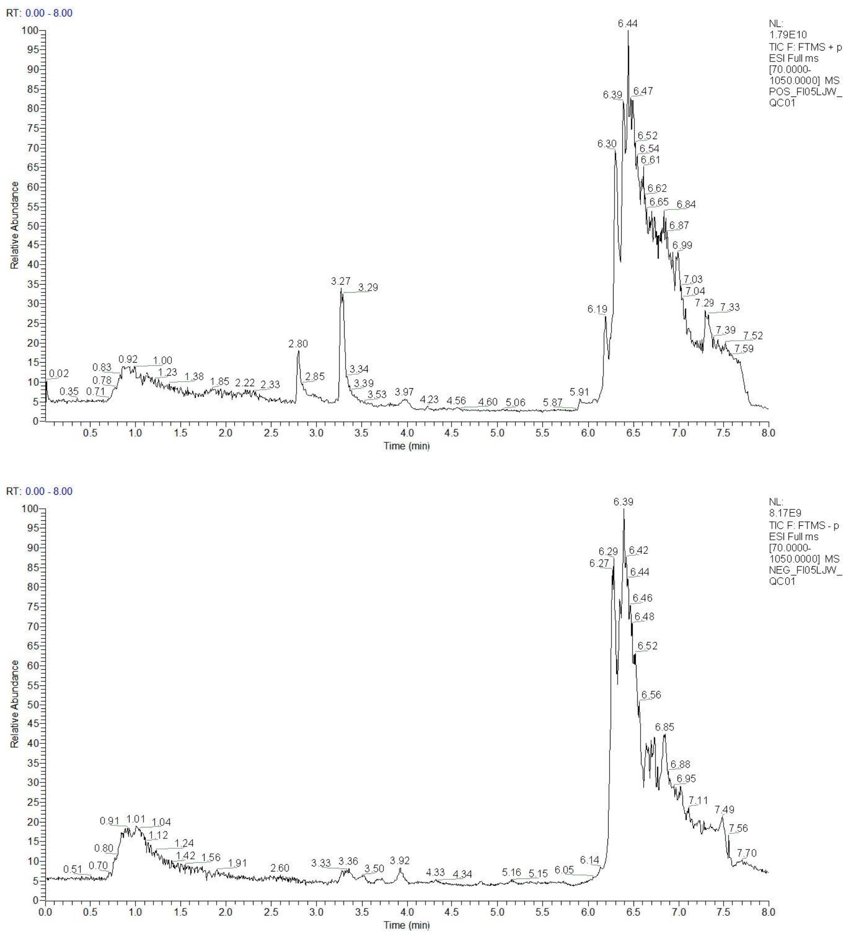

Figure S3. The typical total ions current chromatograms (TICs) of QC samples in positive ion mode (a) and negative ion mode (b).

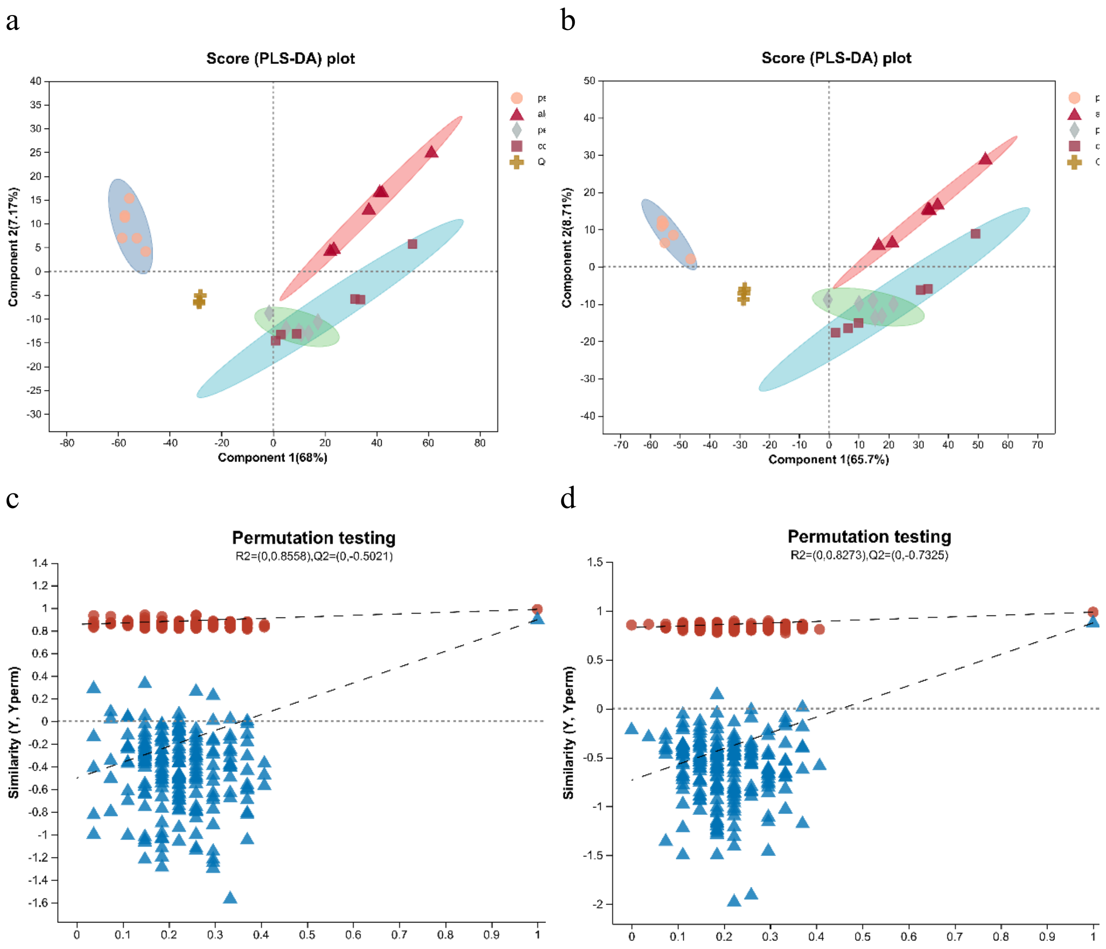

Figure S4. PLS-DA scores and permutation testing of all samples in positive and negative ion mode. (a) PLS-DA scores in positive ion mode; (b) PLS-DA scores in negative ion mode; (c) permutation test in positive ion mode; (d) permutation test in negative ion mode.

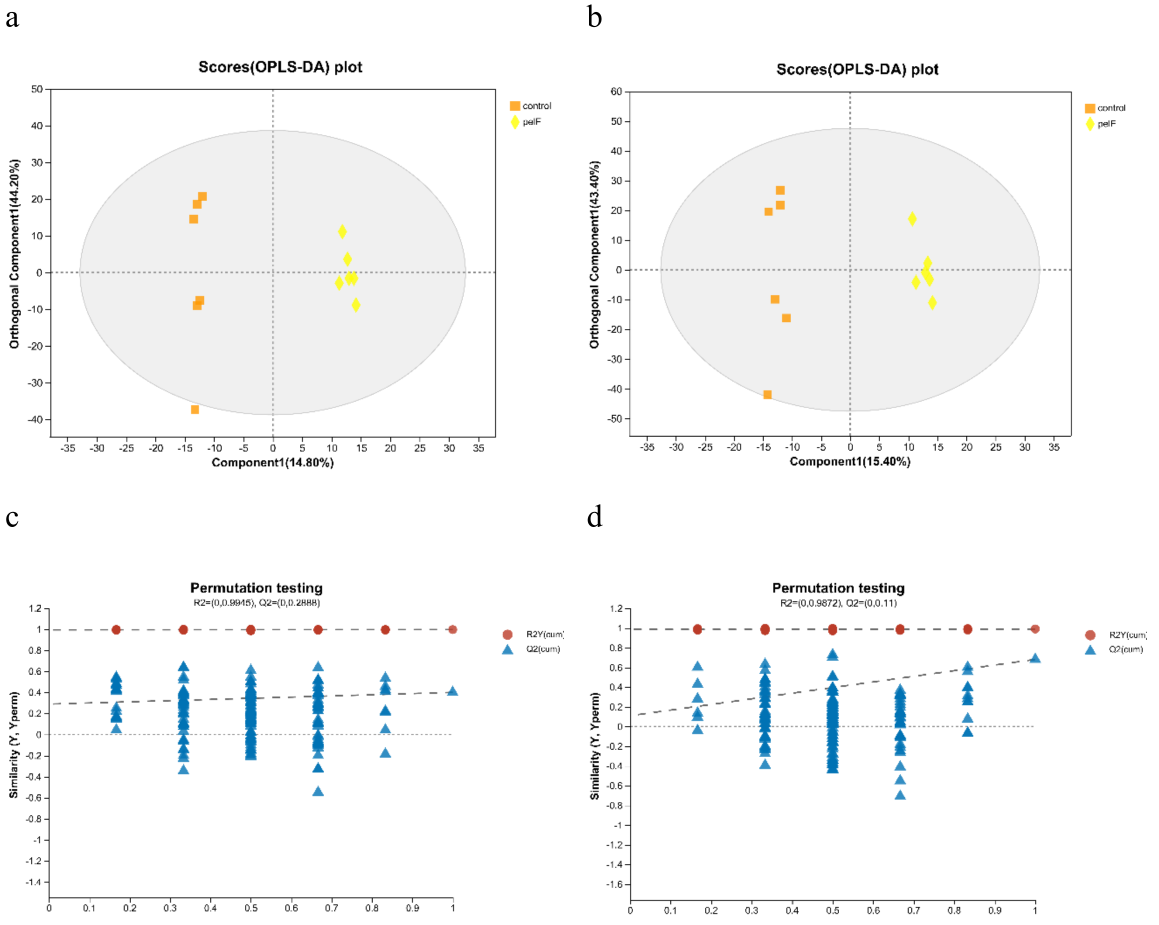

Figure S5. OPLS-DA scores and permutation testing of PAO1-Δ*pelF* vs PAO1-WT in positive and negative ion mode. (a) OPLS-DA scores in positive ion mode; (b) OPLS-DA scores in negative ion mode; (c) permutation test in positive ion mode; (d) permutation test in negative ion mode.

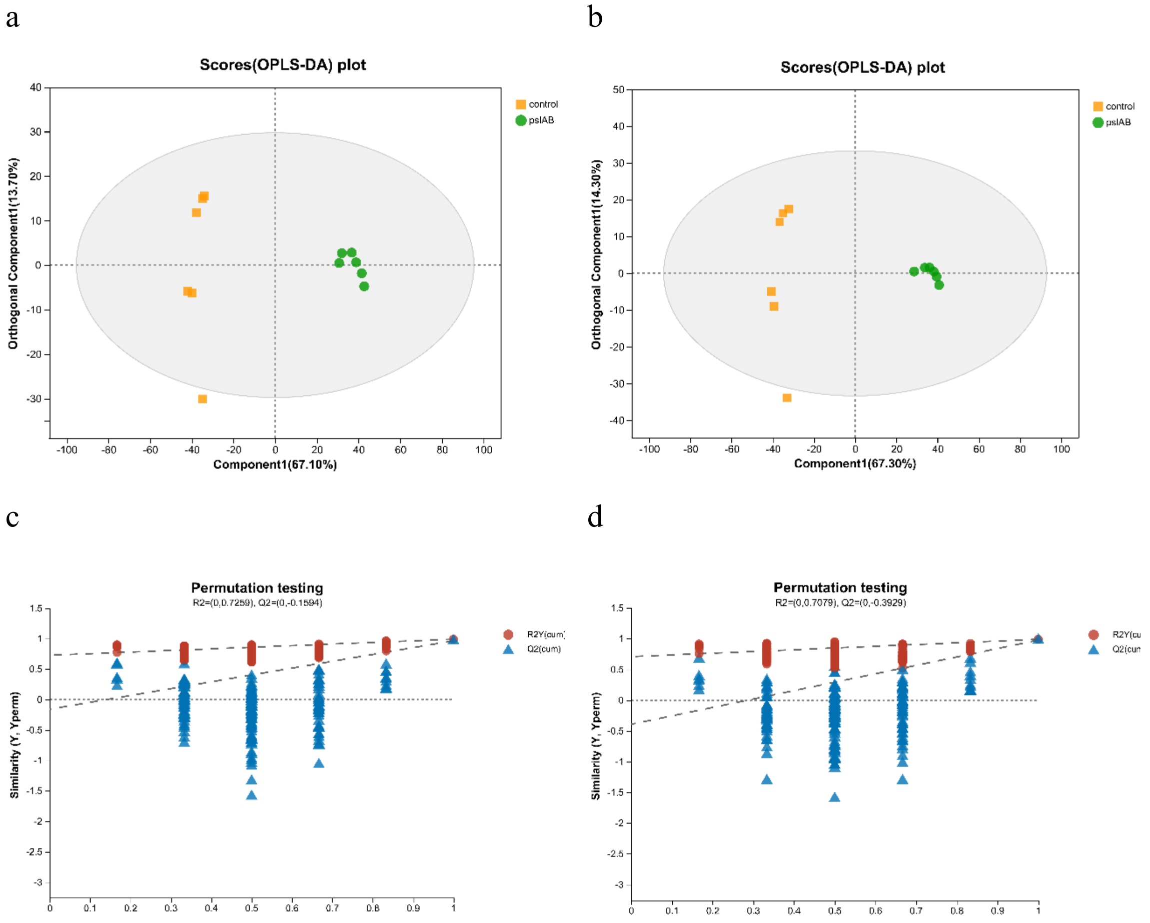

Figure S6. OPLS-DA scores and permutation testing of PAO1-Δ*pslAB* vs PAO1-WT in positive and negative ion mode. (a) OPLS-DA scores in positive ion mode; (b) OPLS-DA scores in negative ion mode; (c) permutation test in positive ion mode; (d) permutation test in negative ion mode.

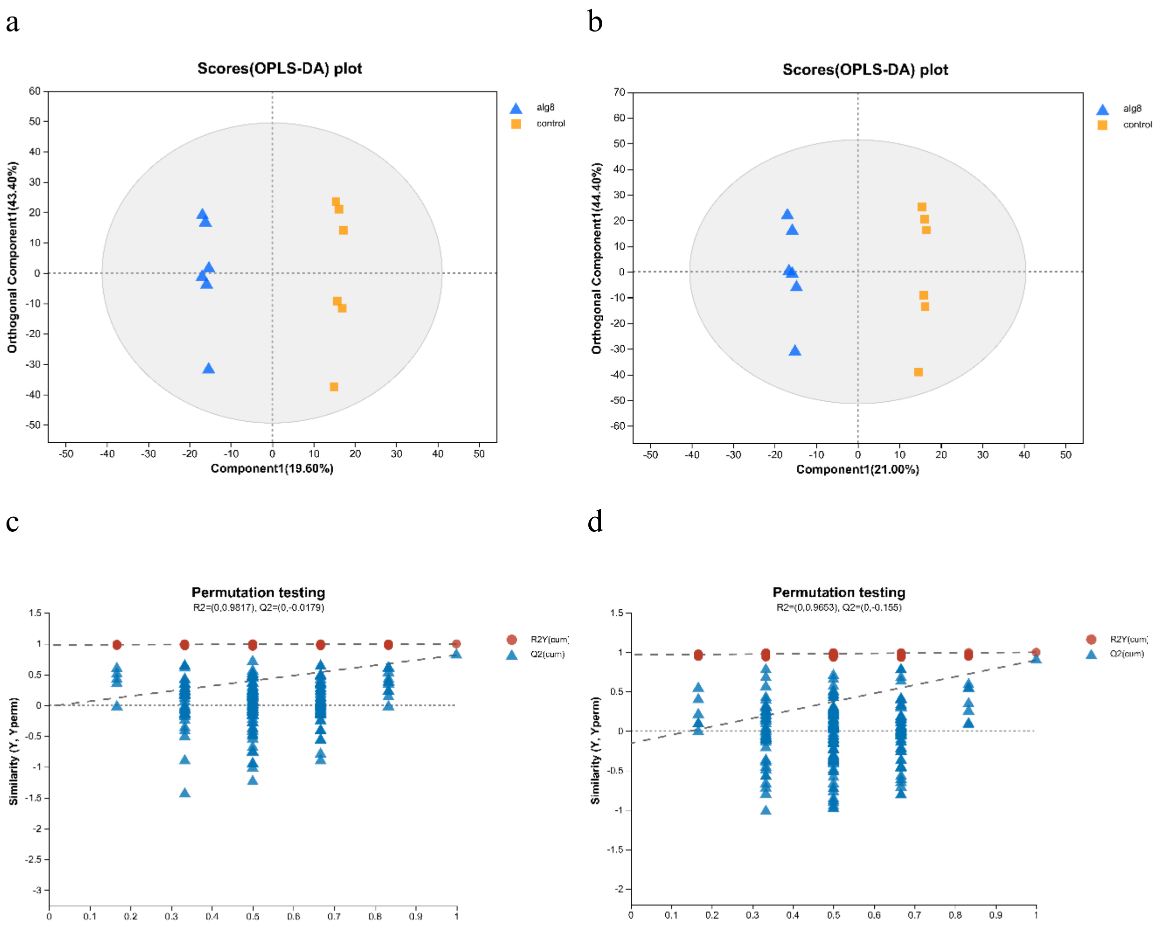

Figure S7. OPLS-DA scores and permutation testing of PAO1-Δ*alg8* vs PAO1-WT in positive and negative ion mode. (a) OPLS-DA scores in positive ion mode; (b) OPLS-DA scores in negative ion mode; (c) permutation test in positive ion mode; (d) permutation test in negative ion mode.

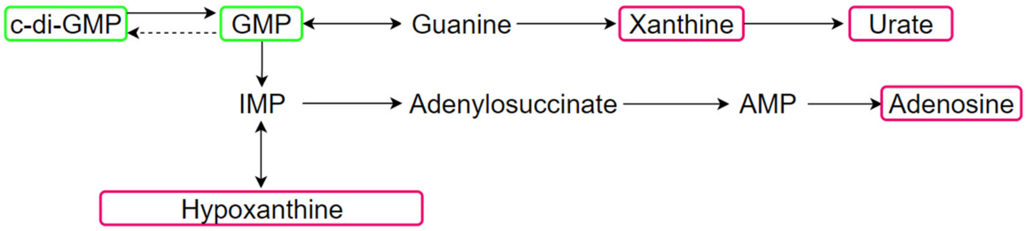

Figure S8. Purine metabolism in PAO1-Δ*pelF* vs PAO1-WT.

Table S1: Biofilm parameters of *P. aeruginosa* PAO1 determined using CLSM.

	Biovolume (10^5 μm^3)	Mean thickness (μm)	Biofilm roughness
WT	10.28±0.23	13.29±2.28	0.21±0.09
Δ*pelF*	7.97±1.11	11.19±2.04	0.22±0.06
Δ*pslAB*	5.15±0.65	8.10±1.16	0.45±0.13
Δ*alg8*	9.77±0.77	13.32±1.51	0.22±0.06

Table S2: Quality test results of RNA.

Sample Name	Concentration(ng/μL)	Amount(μg)	OD260/280	OD260/230	RIN
WT_1	126.90	2.54	2.17	1.88	7.90
WT_2	117.90	2.36	2.15	2.13	8.10
WT_3	122.40	2.45	1.94	1.44	9.40
pelF_1	74.10	1.48	2.13	1.12	7.80
pelF_2	85.80	1.72	2.17	1.45	7.70
pelF_3	69.60	1.39	2.07	1.02	7.90
pslAB_1	971.80	29.15	2.19	2.44	7.30
pslAB_2	1141.40	22.83	2.20	2.34	6.60
pslAB_3	1036.80	20.74	2.17	2.17	7.00
alg8_1	108.60	2.17	2.16	1.76	6.70
alg8_2	97.60	1.95	2.04	1.14	7.30
alg8_3	154.10	3.08	2.05	1.73	9.90

Table S3: Quality control analysis results of clean data.

Sample Name	Raw Bases (bp)	Raw Q20 (%)	Raw Q30 (%)	Clean Bases (bp)	Clean Q20 (%)	Clean Q30 (%)	rRNA Ratio (%)
WT_1	6755524372	96.71	92.98	5477987202	98.06	95.15	0.98
WT_2	5677992600	97.34	93.86	4957470239	98.22	95.31	0.80
WT_3	4625590248	97.16	93.13	4299792404	97.91	94.16	0.32
pelF_1	4175763966	96.88	92.72	3431732716	98.05	94.56	1.45
pelF_2	3592841754	96.25	91.72	3102991786	97.58	93.46	1.40
pelF_3	4169956808	96.76	92.37	3602778171	97.79	93.97	1.15
pslAB_1	4100205076	97.73	94.01	3697157721	98.23	94.8	3.55
pslAB_2	4201706068	97.38	93.35	3757380438	98.11	94.48	2.90
pslAB_3	3765043966	97.43	93.53	3255503404	98.19	94.74	6.00
alg8_1	5396830298	96.56	92.69	4373599068	98.19	95.29	1.33
alg8_2	6490518768	96.4	92.47	5096830845	98.23	95.31	1.11
alg8_3	5272671454	97.17	93.32	4684487215	98.08	94.68	0.27

Table S4: Results of sequence mapping.

Sample Name	Total Reads	Genome Mapped Ratio (%)	Unique Mapped Reads	Unique Mapped Reads Ratio (%)
WT_1	44081434	98.77	41950716	95.17
WT_2	37207788	99.17	35434664	95.23
WT_3	30248584	99.37	29418707	97.26
pelF_1	27296920	98.74	25924027	94.97
pelF_2	23543526	98.97	22449855	95.35
pelF_3	27330468	99.26	26272970	96.13
pslAB_1	26962952	99.38	25483040	94.51
pslAB_2	27612378	99.43	26242621	95.04
pslAB_3	24726914	99.2	22753972	92.02
alg8_1	35171064	98.69	33410995	95
alg8_2	42168358	98.65	39824292	94.44
alg8_3	34395638	99.46	33407153	97.13

Table S5: Some differential genes in PAO1-Δ*pslAB* vs PAO1-WT.

Gene id	Gene name	Gene description	Log_2FC	P adjust	Regulate
PA1246	*aprD*	alkaline protease secretion ATP-binding protein AprD	-1.2767	0.018268374	down
PA1248	*aprF*	alkaline protease secretion protein AprF	-1.4086	0.01071051	down
PA1249	*aprA*	alkaline metalloproteinase	-1.9812	2.48E-07	down
PA4205	*mexG*	hypothetical protein	1.2628	0.019122065	up
PA4206	*mexH*	resistance-nodulation-cell division (RND) efflux membrane fusion protein	1.4750	0.002380612	up
PA4207	*mexI*	resistance-nodulation-cell division (RND) efflux transporter	1.1159	0.021523519	up
PA4208	*opmD*	hypothetical protein	1.4097	0.002107055	up
PA1179	*phoP*	two-component response regulator PhoP	2.3070	5.06E-08	up
PA1180	*phoQ*	two-component sensor PhoQ	2.2200	7.26E-07	up
PA1431	*rsaL*	regulatory protein RsaL	-3.1143	2.64E-17	down
PA5483	*algB*	two-component response regulator AlgB	1.5992	0.000135351	up
PA0764	*mucB*	sigma factor AlgU regulator MucB	1.6366	0.000921788	up
PA0765	*mucC*	positive regulator for alginate biosynthesis MucC	1.6659	0.001753168	up
PA0766	*mucD*	serine protease MucD	1.0518	0.032812066	up

Table S6: PCA model parameters.

A	Positive ion mode		Negative ion mode	
	R^2x	$R^2x(cum)$	R^2x	$R^2x(cum)$
p1	0.556	0.556	0.536	0.536
p2	0.0963	0.652	0.12	0.657
p3	0.0633	0.715	0.0522	0.709

Table S7: PLS-DA model parameters.

Ion mode	A	R^2X(cum)	R^2Y(cum)	Q^2(cum)
Positive	p4	0.809	0.813	0.641
Negative	p4	0.807	0.845	0.693

Table S8: OPLS-DA model parameters.

Sample Groups	A	R^2X(cum)	R^2Y(cum)	Q^2(cum)
Δ*pelF* vs WT (pos)	sum	0.668	0.996	0.4
Δ*pelF* vs WT (neg)	sum	0.665	0.991	0.682
Δ*pslAB* vs WT (pos)	sum	0.808	0.99	0.963
Δ*pslAB* vs WT (neg)	sum	0.816	0.99	0.977
Δ*alg8* vs WT (pos)	sum	0.695	0.998	0.816
Δ*alg8* vs WT (neg)	sum	0.721	0.721	0.899

Table S9: Significantly differential metabolites in PAO1-Δ*pelF* vs PAO1-WT.

Metabolite	Regulate	CAS ID	VIP	FC	P value
3',5'-Cyclic GMP	down	7665-99-8	5.0352	0.5991	2.31E-05

Table S10: Significantly differential metabolites in PAO1-Δ*pslAB* vs PAO1-WT.

Metabolite	Regulate	CAS ID	VIP	FC	P value
Zidovudine monophosphate	up	-	2.393	15.1017	0.001163
Glutaminylhydroxyproline	up	-	1.7421	1.5384	1.43E-07
Adenosine 5'-Monophosphate	up	61-19-8	2.3939	48.1831	0.0005459
(-)-Huperzine A (HupA)	up	-	1.6529	1.5015	1.69E-05
Metkephamid	up	-	1.7623	1.5509	0.0003505
SENECIPHYLLINE	up	480-81-9	1.9607	1.5899	5.14E-06
Imidaprilat	up	89371-44-8	1.5309	1.5892	0.01079
5-Hydroxykynurenamine	up	708-23-6	1.7698	1.5101	1.21E-05
Bryo 1	up	-	1.9161	1.6977	1.39E-07
4-(3-Hydroxy-7-phenyl-6-heptenyl)-1,2-benzenediol	up	158697-56-4	2.1257	2.7121	0.002866
Arg-3-hyp-7-phe-bradykinin	up	-	1.6221	1.5733	0.00798
7alpha-Hydroxy-3-oxo-4-cholestenoate	up	115538-85-7	1.6865	2.2019	0.009304
Alpha-Eleostearic acid	down	506-23-0	1.8185	0.6663	0.002726
(Z)-6-Tetradecene-1,3-diyne -5,8-diol	down	77769-23-4	1.8376	0.6166	4.49E-06
F16	up	-	2.4614	3.8288	9.13E-06
Ganglioside GD3 (d18:1/24:1(15Z))	up	-	1.8785	1.5599	2.16E-08
17beta-Estradiol-2,3-quinone	up	-	1.6954	1.5144	1.22E-05
Ethinamate	up	126-52-3	1.6936	1.5014	3.43E-09
Stercobilinogen	up	17095-63-5	2.077	2.2925	0.003321
PE(PGJ2/P-16:0)	up	-	1.4628	1.693	0.005699
Phe Pro Val	up	-	1.7723	1.589	1.39E-08
CDP-DG(i-22:0/18:2(9Z,11Z))	up	-	1.9653	1.7165	4.76E-09
Tebuconazole	up	107534-96-3	1.7715	1.6621	0.0009427
Thr Pro Phe	up	-	1.6803	1.5013	1.28E-06
N-[(2-methoxyphenyl)methyl] imidazo[1,2-a]pyridine-2-carboxamide	up	-	1.6586	1.5078	5.93E-08
Strictosidine	up	20824-29-7	1.683	1.5747	0.0002493
Ethotoin	up	86-35-1	1.7521	1.5997	5.57E-10
Canesceol	up	82228-15-7	1.6808	1.5181	0.001946
Esculeoside A	up	-	1.8681	1.551	7.19E-08
Eribulin	up	-	1.5876	1.5076	1.30E-05
Cinncassiol D1 glucoside	up	-	1.7102	1.5455	0.0004243
CAMP	up	60-92-4	2.1738	3.6053	0.0002851
Pentanamide	up	626-97-1	1.5622	1.506	0.0002651
L-Homoserine	up	672-15-1	2.0702	1.8288	7.07E-13

Penitrem B	up	11076-67-8	2.1436	1.7647	1.73E-06
Cadaverine	up	462-94-2	2.1348	1.9256	2.04E-09
2-S-glutathionyl acetate	down	10463-61-3	2.4142	0.0024	0.0005573
Val Glu	up	-	1.8454	1.5787	9.16E-07
Teneligliptin	up	-	2.0394	1.9167	2.29E-07
(3Z,5E)-Hepta-3,5-dienoylcarnitine	up	-	1.5574	1.5488	3.99E-06
Proazulene	up	-	1.8826	1.6604	4.95E-09
H-Arg(NO2)-Obzl	up	-	1.856	1.7156	5.41E-06
Nummularine A	up	53947-95-8	2.1261	2.0961	7.80E-05
DIPROTIN B	up	-	1.7013	1.5203	1.03E-05
Rifapentina	up	-	2.1483	1.8891	1.72E-10
Arg-His-Phe-Trp-Gln-Gln	up	-	2.6735	3.5098	3.33E-06
Pinolidoxin	up	152985-39-2	2.4084	2.5333	4.11E-11
Vindoline	up	2182-14-1	1.7514	1.5201	2.24E-06
DIPROTIN A	up	-	1.7922	1.5982	1.38E-06
Cadabicine methyl ether	up	-	1.7971	1.5365	6.75E-09
(R)-Oxypeucedanin	up	3173/2/2	1.8459	1.6365	1.41E-07
Kainic acid	up	487-79-6	1.6956	1.5127	4.48E-05
4-(3-Pyridin-2-yl-[1,2,4] oxadiazol-5-yl)-butyric acid	up	-	1.8154	2.0912	0.006543
CDP-DG(i-15:0/20:5(7Z,9Z, 11E,13E,17Z)-3OH(5,6,15))	up	-	1.8746	1.6109	2.65E-07
Mangalkanyl glucoside	down	259144-63-3	1.883	0.6555	0.00289
Dezocine	up	53648-55-8	1.9661	1.8764	3.56E-10
Urolithin B	up	1139-83-9	1.8174	1.6431	3.39E-06
4-Amino-3-hydroxybutanoylcarnitine	up	-	1.7997	1.5965	6.22E-08
Oxamniquine	up	21738-42-1	1.9662	1.673	1.59E-12
DG(8:0/0:0/5-iso PGF2VI)	up	-	1.862	1.5926	3.32E-08
Val-pro-pro	up	-	1.7572	1.5231	2.91E-05
2-Isopropyl-6-methyl-4-pyrimidone	up	-	1.7516	1.7242	4.09E-06
Protodioscin	up	55056-80-9	1.9013	1.5723	1.20E-09
Artabsin	up	24399-20-0	1.703	1.5146	1.46E-06
Beta-Alanyl-L-lysine	up	-	1.7389	1.5739	3.48E-07
Subaphylline	up	501-13-3	2.1102	2.0592	3.01E-06
Small bacteriocin	up	172617-17-3	2.0687	2.1799	1.37E-05
2,3-Dihydro-6-(3-(2-hydroxymethyl) phenyl-2-propenyl)-benzofuranol	up	-	1.8035	1.5216	4.65E-06

3'-Ketolactose	up	15990-62-2	1.7008	1.5041	6.68E-06
N2-Succinyl-L-ornithine	up	899816-95-6	1.84	1.597	1.18E-08
Morpholine-4-carboxamide	up	-	1.7015	1.5515	5.11E-10
3',5'-Cyclic AMP	up	60-92-4	2.6269	3.0608	5.09E-08
Molybdopterin precursor Z	up	-	1.9243	1.689	2.22E-08
Diethyl hydrogen phosphate	up	598-02-7	1.5878	1.5672	3.36E-06
1-Pyrroline-5-carboxylic acid	up	2906-39-0; 64199-88-8	1.7281	1.5671	2.04E-06

Table S11: Significantly differential metabolites in PAO1-Δ*alg8* vs PAO1-WT.

Metabolite	Regulate	CAS ID	VIP	FC	P value
3',5'-Cyclic GMP	down	7665-99-8	3.623	0.6484	0.0009434

Table S12: Amino acid metabolites of significant enrichment pathway in PAO1-Δ*pslAB* vs PAO1-WT.

Metabolite	Regulate	KEGG Pathway ID	Pathway
Trans-3-hydroxy-L-proline	up	map00330	Arginine and proline metabolism
L-Lysine	up	map00310; map00300;	Lysine degradation; Lysine biosynthesis
2-Aminomuconic acid semialdehyde	up	map00380;	Tryptophan metabolism
2-Keto-6-acetamidocaproate	up	map00310	Lysine degradation
5-Hydroxykynurenamine	up	map00380	Tryptophan metabolism
Saccharopine	up	map00310; map00300	Lysine degradation; Lysine biosynthesis
Indoxyl	up	map00380	Tryptophan metabolism
5-Acetamidovalerate	up	map00310	Lysine degradation
Tryptophol	up	map00380	Tryptophan metabolism
N6-Acetyl-L-lysine	up	map00310	Lysine degradation
4-Acetamidobutanoic acid	up	map00330	Arginine and proline metabolism
Serotonin	up	map00380	Tryptophan metabolism
Linatine	up	map00330	Arginine and proline metabolism
Diaminopimelic acid	up	map00300	Lysine biosynthesis
5-Hydroxy-L-tryptophan	up	map00380	Tryptophan metabolism
Pipecolic Acid	up	map00310	Lysine degradation
L-Glycine	up	-	Lysine degradation; Purine metabolism; Glutathione metabolism
Creatinine	up	map00330	Arginine and proline metabolism
L-Homoserine	up	map00300	Lysine biosynthesis
Cadaverine	up	map00310; map00480	Lysine degradation; Glutathione metabolism
L-Aspartate-semialdehyde	up	map00300; map00330;	Lysine biosynthesis; Arginine and proline metabolism
Ornithine	up	map00220; map00330; map00480	Arginine biosynthesis; Arginine and proline metabolism; Glutathione metabolism
Acetyl-CoA	down	-	Lysine degradation; Glutathione metabolism; Lysine biosynthesis; Tryptophan

			metabolism; Propionate metabolism
Kynurenic Acid	up	map00380	Tryptophan metabolism
Formyl-5-hydroxykynurenamine	up	map00380	Tryptophan metabolism
6-Hydroxymelatonin	up	map00380	Tryptophan metabolism
Subaphylline	up	map00330	Arginine and proline metabolism
Glutaric Acid	up	map00310	Lysine degradation
Oxoadipic acid	up	map00310; map00300; map00380	Lysine degradation; Lysine biosynthesis; Tryptophan metabolism
N2-Succinyl-L-ornithine	up	map00330	Arginine and proline metabolism
1-Pyrroline-5-carboxylic acid	up	map00330	Arginine and proline metabolism

Table S13: Differential genes related to ferric ion intake in PAO1-Δ*pslAB* vs PAO1-WT.

Gene id	Gene name	Gene description	Log_2FC	P adjust	Regulate
PA0051	*phzH*	phenazine-modifying protein	1.1811	0.023961206	up
PA4217	*phzS*	hypothetical protein	1.2030	0.022396428	up
PA4221	*fptA*	Fe(III)-pyochelin outer membrane receptor	-1.5922	0.000681849	down
PA4224	*pchG*	pyochelin biosynthetic protein PchG	-1.9079	2.48E-07	down
PA4225	*pchF*	pyochelin synthetase	-1.9899	0.019122065	down
PA4226	*pchE*	dihydroaeruginoic acid synthetase	-1.9128	0.002380612	down
PA4228	*pchD*	2%2C3-dihydroxybenzoate-AMP ligase	-2.7301	0.021523519	down
PA4229	*pchC*	pyochelin biosynthetic protein PchC	-2.6757	0.002107055	down
PA4230	*pchB*	isochorismate-pyruvate lyase	-2.4686	5.06E-08	down
PA4231	*pchA*	salicylate biosynthesis isochorismate synthase	-2.6186	7.26E-07	down
PA2386	*pvdA*	L-ornithine N5-oxygenase	-1.9078	0.000964659	down
PA2396	*pvdF*	pyoverdine synthetase F	-1.2772	0.024462117	down
PA2397	*pvdE*	pyoverdine biosynthesis protein PvdE	-2.0506	9.54E-05	down
PA2398	*fpvA*	ferripyoverdine receptor	-1.5053	0.005242665	down
PA2399	*pvdD*	pyoverdine synthetase D	-2.4120	2.26E-06	down
PA2400	*pvdJ*	pyoverdine biosynthesis protein PvdJ	-2.6237	1.42E-07	down
PA2413	*pvdH*	diaminobutyrate--2-oxoglutarate aminotransferase	-1.7510	0.002557302	down
PA2424	*pvdL*	peptide synthase	-2.4201	7.16E-06	down
PA2425	*pvdG*	pyoverdine biosynthesis protein PvdG	-1.9606	6.08E-05	down
PA2686	*pfeR*	two-component response regulator PfeR	1.1498	0.043148795	up
PA2687	*pfeS*	two-component sensor histidine kinase PfeS	1.6431	0.004480439	up
PA2688	*pfeA*	ferric enterobactin receptor	1.3997	0.021175138	up
PA4159	*fepB*	iron-enterobactin transporter periplasmic binding protein	1.6290	0.001082635	up
PA4168	*fpvB*	second ferric pyoverdine receptor FpvB	-1.3448	0.030163811	down

PA4221	*fptA*	Fe(III)-pyochelin outer membrane receptor	-1.5922	0.000681849	down
